# Effects and regulation of ACE2 and TMPRSS2 abundance in healthy humans and in patients with SARS-CoV-2

**DOI:** 10.1042/BST20241052

**Published:** 2025-07-08

**Authors:** Marie Lykke Bach, Boye L. Jensen

**Affiliations:** Unit of Cardiovascular and Renal Research, Department of Molecular Medicine, University of Southern Denmark, Campusvej 55, Odense 5230 Odense M, Denmark

**Keywords:** circulation, COVID, hypertension, kidney, renin, shedding

## Abstract

The present narrative review focuses on organ distribution, co-localization, age-, and sex-dependent changes in angiotensin-converting enzyme 2 (ACE2) and transmembrane serine protease 2 (TMPRSS2) and how such changes associate with SARS-CoV-2 virus entry and disease severity in humans. ACE2 is a membrane-bound enzyme with lower abundance in children/young adults compared with elderly, with no protein abundance difference between ages 35–50 and >80 but higher in females at reproductive age. ACE2 locates predominantly in gastrointestinal (GI)-tract epithelia, kidney proximal tubules, male and female reproductive organs with very low levels in the lungs. Estrogen upregulates ACE2, which can be shed from cells into plasma by, for example ADAM17, while remaining active. TMPRSS2 is a membrane-associated serine protease with androgen dependence. The highest levels in humans are found in male reproductive organs, kidney, and GI-tract. Co-localization with ACE2 in alveolar type 2 cells is based mostly on *in vitro* studies. Documentation of clustering of ACE2 and TMPRSS2 in human tissues is scarce and best in oral-pharyngeal mucosa. In patients with mild-to-serious COVID-19 disease, there is no consistent change in circulating renin, aldosterone, ACE and ACE2 activities, angiotensin II (ANGII), and Ang1–7. Increased ANGII levels are reported in critically ill patients, while ACE2 is massively present in urine. Use of RAAS inhibitors is not associated with negative outcomes in patients with COVID-19. In conclusion, co-localization of ACE2 and TMPRSS2 in oral and airway epithelia may explain the primary route of infection for SARS-CoV-2 virus. Higher risk for serious disease in elderly males may not be accounted for by quantitative changes in the proteins.

## Introduction

The COVID-19 pandemic, caused by the novel coronavirus SARS-CoV-2 that emerged in 2019, has led to more than 700,000,000 confirmed cases globally [1]. The response to SARS-CoV-2 infection depends on demographic factors of the individual, viral load, and co-morbidities such as hypertension, chronic kidney disease, and diabetes mellitus, which all predict severe COVID-19 disease [2–4]. While male and female sexes show an equal prevalence of COVID-19, epidemiological data show a sex-dependent clinical difference in SARS-CoV-2 severity with a higher mortality rate in male patients and elderly patients [5–8]. Thus, male sex and advanced age are established as significant risk factors of severe COVID-19 disease explained, at least in part, by the higher frequency of co-morbidities such as cardiovascular disease [9–11]. Cell-surface-bound angiotensin-converting enzyme 2 (ACE2) with adjacent protease transmembrane serine protease 2 (TMPRSS2) is an established route of cellular entry for SARS-CoV-2 in lung epithelia [12–14]. There, ACE2 serves as a receptor for the spike glycoprotein of SARS-CoV-2, which is subsequently cleaved by the proteolytic activity of TMPRSS2, facilitating virus-cell membrane fusion and entry [15–17]. Also, cysteine proteases cathepsin B/L (CatB/CatL) may mediate entry [18], and five serine proteases, including TMPRSS2, TMPRSS13, TMPRSS11D, TMPRSS11E, and TMPRSS11F, significantly facilitate SARS-CoV-2 entry [19], while three of six membrane-type matrix metalloproteinases (MMP14, MMP16, and MMP17) and four of eight ADAMs (ADAM8, ADAM9, ADAM12, and ADAM33) also mediate SARS-CoV-2-S pseudo virus entry *in vitro*, albeit not as efficiently when compared with serine proteases, including furin [14,20]. SARS-CoV-2 variants employ different protease preferences [19], and while the presence of ACE2 appears as necessary and limiting for entry, there is more redundancy regarding available cell-surface-bound proteases. ACE2 and TMPRSS2 proteins display a wide tissue distribution and are abundant in epithelial tissue (e.g., lung, kidney, gastrointestinal [GI] tract), but whether the proteins co-localize and facilitate viral uptake beyond lungs is less clear. The question is relevant since ACE2 shows much higher expression in GI and kidney epithelia than in the lung [21]. The pathophysiological systemic consequences of viral engagement with ACE2 with respect to changes in substrate concentration, angiotensin II (ANGII), and catalytic product, angiotensin 1–7 (Ang1–7), are not consistent. The present narrative review aims to elucidate and summarize data on organ-specific distribution of ACE2 and TMPRSS2 with emphasis on the link between abundance, localization, and susceptibility to COVID-19; the changes that occur with age and with special focus on differences between sexes in relation to susceptibility and pathophysiology including circulating peptides in patients with SARS-CoV-2 infection.

### Scope

The authors have focused on original studies and studies in humans. Studies with patients were included only if there were control groups and well-described criteria for assays used to detect components in plasma and tissue. The authors have cited pre-clinical animal studies only when mechanistic insights not otherwise possible to obtain were reached (genetic manipulations, drug candidate infusions, etc.). The authors have no commercial or private financial interests in diagnostics, therapeutics, or preventive measures in relation to patients with COVID-19 or other diseases. The search strategy keywords were COVID-19, SARS-CoV-2, ACE2, TMPRSS2, protein, level or concentration, age-aging, female-male, and filters ‘human’.

### Organ distribution and cellular co-localization of key proteins ACE, ACE2, and TMPRSS2

ACE2 was discovered in 2000 [22,23] in the kidney, while its isoform ACE was identified functionally in the lung in 1956 by Skeggs et al. [24]. Both proteins are zinc metalloproteases involved in the renin-angiotensin-aldosterone system (RAAS) that share a similar structure but with distinct effects. ACE consists of two domains: N-terminal and C-terminal with dual enzymatic activity, facilitating the conversion of ANGI to the vasoconstrictor ANGII. The C-terminal of ACE2 is a short cytoplasmic tail with no enzymatic activity, while the extensively N-glycosylated N-terminal ectodomain contains the active site. Membrane-bound ACE2 degrades several angiotensin peptides with the highest affinity to ANGII, which is hydrolyzed to vasodilatory peptide Ang1–7 and considered part of the ‘protective’ arm of the RAAS increasing nitric oxide formation through the Mas receptor [25]. ACE and ACE2 proteins are present across various organs in the human body [26]. Integrating publicly available mRNA and protein maps and databases show that ACE2 is by far most abundant in kidney proximal tubular cells and GI epithelia in humans, whereas lung alveolar type 2 cells (AT2) show orders of magnitude lower levels of ACE2 protein [21,26,27]. ACE protein and mRNA abundance is greater than ACE2 in lungs, and as for ACE2, ACE displays the highest levels in the kidney proximal tubular cells and GI epithelial cells [21]. In the kidney, the two proteins co-localize in the apical brush border of the proximal tubular cells, where they act simultaneously and tentatively with substrates in series (ANGI→ANGII→Ang1–7). ANGI and ANGII are deca- and octapeptides and thus filtered freely from plasma across the glomerular barrier. Both peptides can be detected in urine in small quantities as the peptides are extracted from the ultrafiltrate via apical degradation and/or megalin-dependent endocytosis [28,29]. At the systemic level, the ACE and ACE2 enzymes are predicted to have opposing roles in blood pressure regulation. The membrane-bound forms of ACE2 and ACE can be shed from cells by proteolytic activity by, for example, ADAM17, but can also be part of extracellular vesicles released by all cells. Ectodomain shedding releases catalytically active soluble forms of ACE2 and ACE into circulation [30,31]. TMPRSS2 belongs to the type-2 transmembrane serine protease family involved in proteolytic remodeling of the extracellular matrix (ECM). TMPRSS2 is composed of an intracellular domain, a single-pass transmembrane domain, and a biologically active ectodomain with three subdomains: a low-density lipoprotein receptor type-A domain, a Class A Scavenger Receptor Cysteine-Rich domain, and a C-terminal trypsin-like serine peptidase domain with a canonical Ser441–His296–Asp345 catalytic triad [32,33]. As TMPRSS2 is synthesized as a zymogen, it undergoes autocleavage to reach its mature active form. The mature catalytic chain of TMPRSS2 migrates at 24–35 kDa in SDS-PAGE, dependent on the expression system [34,35]. The protease is widely distributed in human epithelial tissue both on protein and mRNA level [21,36,37]. The physiological function of TMRPSS2 *in vivo* remains elusive, but it is part of the extracellular matrix, and in the kidney, it can cleave and activate the epithelial sodium channel [35,38,39].

### Physiological regulation of ACE2 and TMPRSS2 by sex hormones

The abundance of TMPRSS2 is promoted by androgens—testosterone and dihydrotestosterone, particularly in the prostate epithelium [34,38,40]. TMPRSS2 is involved in carcinogenesis of prostatic cancer, promoting cancer cell invasion and metastasis by stimulating epithelial-to-mesenchymal signaling, degradation of the ECM, and fusion with the oncogenic transcription factor ERG [40–42]. However, there is no indication that androgens affect TMPRSS2 expression in lung, GI, and kidney tissue [43]. Estrogen increases circulating levels of renin-substrate, angiotensinogen, in post-menopausal women [44–46]. Chronic low infusion of ANGII decreased arterial pressure by 10 mmHg in female rats at a rate that increases pressure in male rats, emphasizing the cardiovascular ‘protective’ role of estrogen [47,48]. In humans, right atrial appendage tissue from patients undergoing heart surgery showed ACE2 mRNA level correlated positively with exposure to estrogen and the estrogen receptor alpha [49,50]. Similarly, the transcript levels of TMPRSS2 and ADAM17 correlated with estrogen receptor alpha levels in the human right atrium [50]. In summary, reproductive epithelia and some cardiovascular tissues show sex-specific stimulatory effects of steroids such that ACE2 is supported by female steroids and TMPRSS2 by male counterparts.

### Co-localization of ACE2 and TMPRSS2

**Figure 1:**
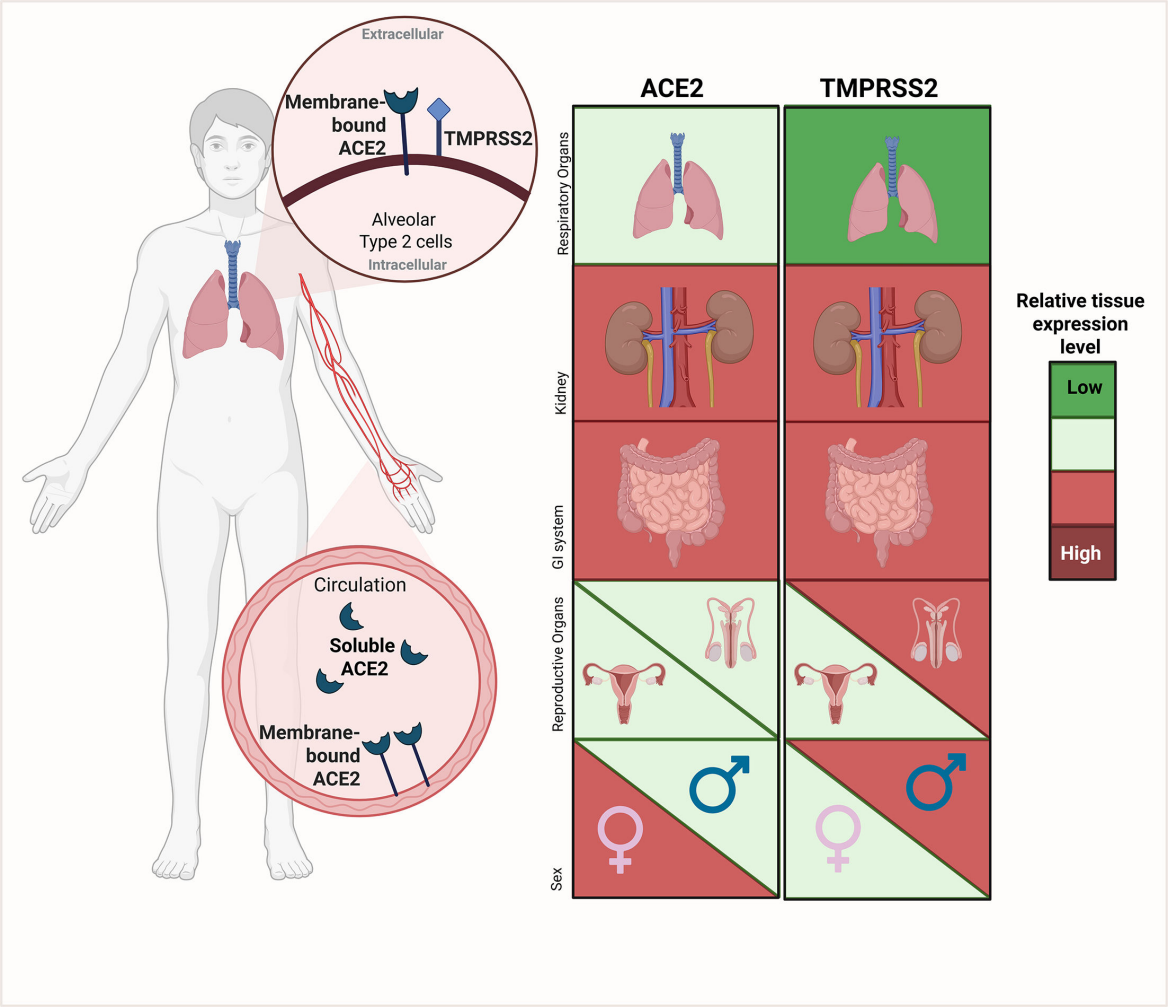
ACE2 and TMPRSS2 levels in lungs, kidney, gastrointestinal (GI) tract, reproductive organs, and how individual sex affects the levels. Protein/mRNA levels are illustrated by a green-to-red color scale. Green is low and red is high. The two proteins co-localize in alveolar type 2 cells in the lung while cellular clustering is less clear in other differentiated cell types. In the circulatory system ACE2 is expressed in endothelial cells and it can be detected in soluble form in plasma. In healthy tissue, the level of ACE2 is higher compared with TMPRSS2 in the lung and is unchanged with age in adults while showing lower levels in children. In the kidney, ACE2 abundance is many times higher than in the lung and does not change with age in adults. ACE2 level is stimulated by estrogen which accounts for slightly higher levels in females in reproductive age. TMPRSS2 is regulated by androgens. The level of TMPRSS2 in the male reproductive organs is higher compared with female reproductive organs. Kidneys show high levels of TMPRSS2 and here, the level is unchanged with age. The level of ACE2 and TMPRSS2 is high in the GI system, similar to kidneys and many times higher than in lungs. In the GI tract with increasing age the level of ACE2 increases while TMPRSS2 remains the same. Created in BioRender. Bach, M. (2025) https://BioRender.com/0fqsfye.

It is striking that the profile of TMPRSS2 localization and abundance in human tissues does not match that of ACE2—at least not at sites believed to be crucial for the entry for the SARS-CoV-2 virus. Single-cell analysis of human heart reveals high expression of ACE2 while TMPRSS2 is scarcely expressed [51]. RNA-seq reveals co-expression of ACE2 and TMPRSS2 in healthy lung tissue AT2 cells, considered the primary site of infection [52]. To our knowledge, data at protein level are less clear. Based on Pearson’s correlation test, ACE2 and TMPRSS2 co-expressed at a higher degree compared with ACE2 and, e.g., CatB/L and furin [53]. In the kidney, ACE2 is expressed primarily in the proximal tubules, whereas TMPRSS2 is detected predominantly in the distal part of the nephron [54–56]. Staining of healthy kidney tissue by immunohistochemistry does not indicate co-localization [57]. Observations support that lung AT2 cells have ACE2 and TMPRSS2, while in other tissues, data on co-localization are inconsistent. In summary, ACE2 and TMPRSS2 display disparate organ distribution with some overlap in the GI tract, reproductive organs, kidneys, and lung AT2 cells, and while ACE2 is stimulated by estrogen, TMPRSS2 is androgen-dependent (Figure 1). Since viruses enter human host cells upon binding to ACE2, the GI tract, kidneys, and reproductive organs would appear the most susceptible to COVID-19 infection. A priori, female gender would be most susceptible based on ACE2 abundance, while males would seem more at risk based on TMPRSS2. Do data from humans allow conclusions as to whether variable susceptibility to SARS-CoV-2 with age (children less than elderly, young adults less than elderly) and sex (women less susceptible than men) relates to changes in abundance of ACE2 and TMPRSS2 across age, sex, and co-morbidities?

### Sex and age-related changes in TMPRSS2 and ACE2 in healthy persons and differences in susceptibility to COVID-19

SARS-CoV-2 transmission occurs primarily by respiratory route through airborne droplets and close contact. The relative importance of airborne, droplet, swallowed particles, and plasma-borne and fomite transmission routes for SARS-CoV-2 depends on the co-presence of ACE2 serving as receptor and TMPRSS2 activity in organs. In serum, large population studies show that subjects with higher risk for severe COVID-19 had higher soluble ACE2 (sACE2) (adults > children and male > female) [58]. Quantitative data on ACE2 and TMPRSS2 proteins in tissues are scarce, whereas data on mRNA are more available. In the following, our focus has been to provide an overview of available data from humans.

Whereas fetal human kidneys have cells expressing both ACE2 and TMPRSS2 [59], children displayed lower ACE2 in lung tissue compared with young and elderly adults by immunohistochemistry [60]. The amount of ACE2-expressing AT2 cells and ACE2 protein content were lower in children than in adults. The lower levels of ACE2 observed in children in the respiratory organs may partially explain their lower susceptibility to COVID-19. These observations in children support that lower levels of ACE2 are protective against SARS-CoV-2 entry.

**Figure 2:**
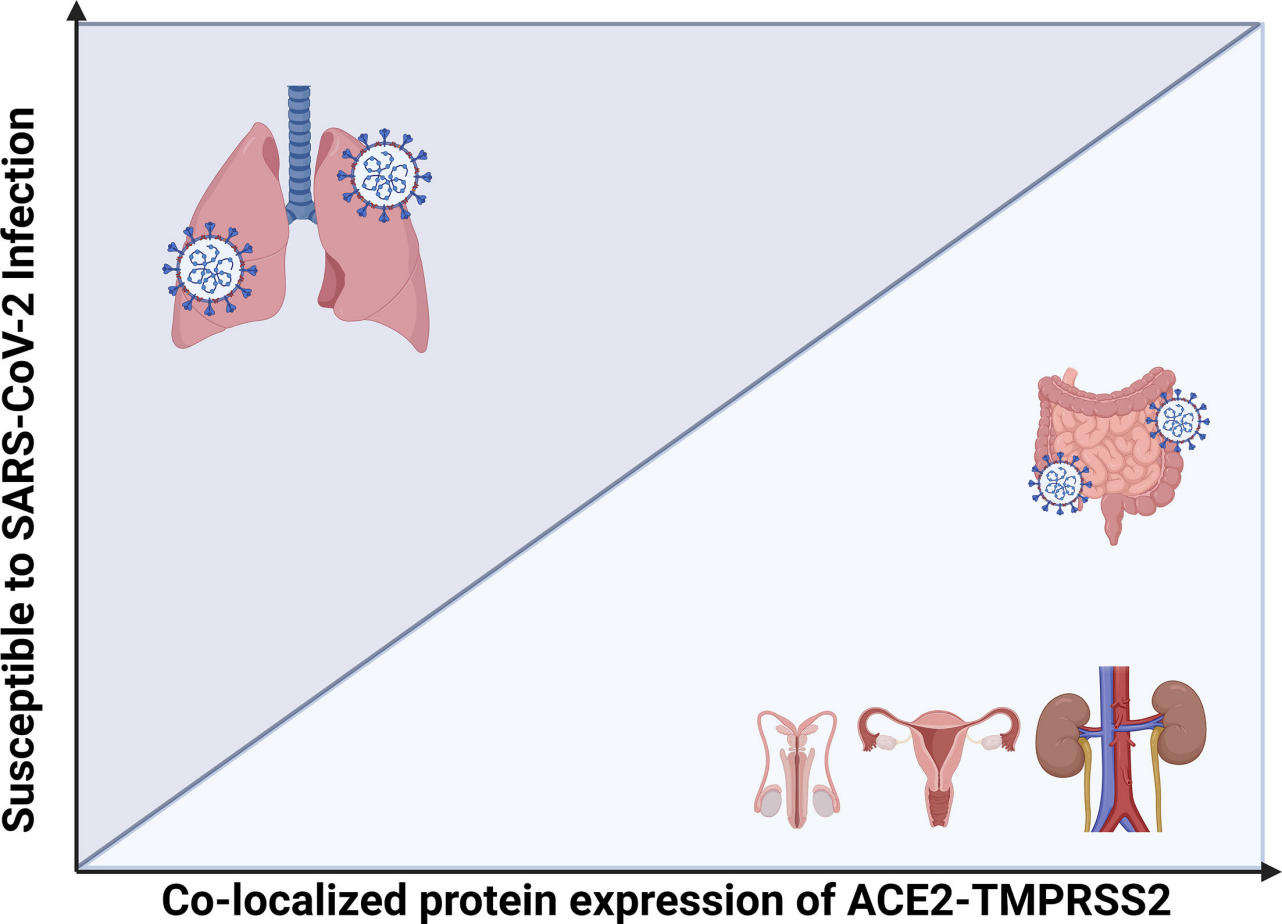
Relation between tissue susceptibility to SARS-CoV-2 infection and ACE2/TMPRSS2 protein co-localization and level. Susceptibility to SARS-CoV-2 infection (Y-axis) is plotted against the level of ACE2 and TMPRSS2 co-localization (X-axis) across multiple ‘epithelial’ organ systems. The respiratory organs are highly susceptible to SARS-CoV-2 infection but have low level of ACE2 and TMPRSS2 co-localization, while the kidney and reproductive organs have high expression level of ACE2 and TMPRSS2 but have low susceptibility to SARS-CoV-2 infection. The gastro-intestinal tract has high expression of the two proteins and moderate susceptibility to SARS-CoV-2 infection. Created in BioRender. Bach, M. (2025) https://BioRender.com/h29i912.

Which data are available for adults? In patient duodenal biopsies, ACE2 mRNA level correlated with age [61]. In a large-scale transcriptomic approach across three independent datasets of human kidney tissue (*n* = 720 individuals) combining whole tissue transcriptome profiling but also single-cell investigations, ACE2 mRNA is highly abundant in the proximal tubule with a clear direct relation with sex and age; however, with no association with hypertension or use of RAAS drugs. In the combined analysis of 534 samples, females had ~1.4-fold higher levels of renal ACE2 expression when compared with males [62]. As in the above study, our group selected the human kidney as a tissue of key importance to understand age- and sex-related changes in ACE2 and TMPRSS2. A large biobank of freshly frozen healthy tissue from cancer-nephrectomy from human adult patients allowed quantitative analyses of protein levels for ACE2 and TMPRSS2. By immunoblotting and ELISA, ACE2 showed no change in protein abundance with high age (above 80) compared with ages below 50 years. Across age groups, ACE2 protein abundance was slightly but significantly higher in females compared with males. In the oldest subgroup above 80 years, there was no difference in ACE2 abundance between sexes, and there was no difference in protein abundance of TMPRSS2 in kidneys. By contrast, when analyzing urine extracellular vesicles, reflecting cell surface-bound proteins, TMPRSS2 was higher in vesicles from males [57]. In summary, two larger studies on human kidney tissue agree that ACE2 is slightly more abundant in females than males, but the difference disappears in older age. In a study of oral mucosa tissue, ACE2 and TMPRSS2 were higher in elderly patients, and ACE2 was higher in females than males and vice versa for TMPRSS2 [63]. Cardiac tissue from explanted hearts showed elevated ACE2 protein levels in aged males compared with aged females by immunofluorescence. ACE2 activity was consistent with the immunoblot results [64]. Based on ACE2 and TMPRSS2 healthy tissue levels, organs can be grouped into susceptibility levels to SARS-CoV-2 infection (Figure 2) [65]. The respiratory system is the primary site of infection. However, the GI tract has high expression of ACE2 and TMPRSS2 as well, where some patients experience GI symptoms without any respiratory symptoms in the initial stages of the disease [66]. Similarly, the kidney has a high expression level of ACE2 and TMPRSS2 and is considered susceptible to infection (Figure 2). Indeed, acute kidney injury is associated with severe COVID-19, but the detection of virus protein and RNA in urine and in kidney tissue is not consistent, and few data show parenchymal infection [67–71]. A recent study could not detect SARS-CoV-2-related RNA by polymerase chain reaction in purified urine extracellular vesicles from patients with severe COVID-19. Nor could ACE2 and TMPRSS2 be co-localized by immunoprecipitation of urine extracellular vesicles [57]. Thus, organ systems with high expression of ACE2 with proven changes with sex do not fully match the clinical observations of morbidity and mortality. Moreover, the increased susceptibility to infection, particularly in elderly males, does not correspond with protein level. Data support that children exhibit lower levels of ACE2 at the primary entry route and have less severe disease.

### Changes in plasma and urine ACE2 in COVID-19 and role of ACE2 shedding and ADAM17 activity for susceptibility toward COVID-19

If organ levels cannot account for enhanced susceptibility and negative outcomes in elderly males, could other COVID-19 induced changes in ACE2 and downstream products account for this? Circulating ACE2 levels have been compared between children and adults and related to sex. In adults and children with COVID-19, serum ACE2 concentrations were elevated compared with healthy individuals. Serum ACE2 levels increased with age, with low levels in children but no change with sex [72]. Elevated circulating ACE2 was affirmed in patients with COVID-19 compared with healthy controls [73], and ACE2 concentration was associated with negative clinical outcomes in patients with COVID-19 disease [74]. Other studies report no difference in serum ACE and ACE2 concentrations between patients with COVID-19 compared with matched controls [75], while studies find that patients admitted with COVID-19 display lower ACE and no change in ACE2 activity [76]. Thus, data from patients indicate null to modestly increased release or shedding of ACE2 associated with age and with COVID-19. What is the mechanism? Does the increase in immunogenic circulating ACE2 also reflect ACE2 activity, i.e., is released or shedded ACE2 catalytically active? Differences observed in some studies with COVID-19 infected patients could have other reasons since hypertension and diabetes are the most frequent co-morbidities, and these diseases are associated with elevated soluble ACE2 in plasma and thus must be considered significant confounders [77,78]. If patients with hypertension and diabetes are infected, significantly higher plasma levels of ACE2 are associated with disease severity [79]. A candidate enzyme ADAM17 that releases membrane-bound ACE2 has increased activity in systemic infection [80,81] and also by various ‘stressors’ such as high glucose [82] and ANGII [83]. With no change to modest increase in sACE2, can changes be seen in urine since kidneys have high expression of ACE2? Our group identified 250-fold increases in ACE2 urine/creatinine ratios from patients with COVID-19 disease compared with healthy control and relating directly to albumin in urine but beyond what could be explained by aberrant glomerular filtration. This indicates a combination of increased shedding of ACE2 and aberrant filtration [57]. In patients, acute kidney injury and COVID-19 correlated with elevated urine ACE2 [84]. Does shedding of ACE2 increase susceptibility to SARS-CoV-2 infection or rather decrease it? SARS-CoV-2 can bind several soluble ACE2 molecules forming virus receptor complexes, saturating the virus binding sites, making the virus unable to fuse with the cell membrane and protecting from viral infiltration [85]. Thus, recombinant ACE2 presents a potential therapeutic approach against COVID-19, best documented in animal experiments, leveraging its role as a viral decoy receptor to disrupt SARS-CoV-2 attachment to human cells. A case report suggests beneficial effects of ACE2 infusion in a critically ill patient with subsequent decrease in plasma ANGII and increase in Ang1–7 peptides delivering proof-of-concept [86], but clinical trials are essential to elucidate its efficacy, optimize delivery methods, and confirm safety in diverse patient populations. To our knowledge, no clinical trials have tested the effect of Ang1–7 delivery.

On the other hand, circulating virus-bound ACE2 may become unmeasurable, accounting for studies that measure lower or no change in ACE2 plasma levels. Whether plasma ACE2 concentration *per se* plays a significant role in pathophysiology and susceptibility in COVID-19 is unclear, but changes in ACE2 concentration and/or activity predict the potential systemic involvement of changes in angiotensin concentrations. Moreover, it leaves the question: What is more significant for the progression of severe disease, loss of ACE2/Ang1–7 and/or increase in ANGII?

### Changes in circulating levels of angiotensin peptides in patients with COVID-19 infection

Some circulatory pathophysiological traits (hypertension, endothelial dysfunction) in patients with severe COVID-19 were rapidly linked to RAAS changes, as the balance between the two arms (ACE-ANGII-Angiotensin II Type 1 receptor (AT1R) versus ACE2-Ang1–7-Mas receptor) of the system could change significantly if ACE2 abundance or activity is lowered by SARS-CoV-2 infection and renin secretion increased. Pre-pandemic, pre-clinical observations on the SARS virus showed that ACE2 mRNA abundance was downregulated in infected mouse lungs, that the spike protein down-regulated surface ACE2, that experimental, acid-induced, lung injury was augmented with infection and, notably, that lung tissue ANGII content increased *in vivo* with experimental injury, which was attenuated by ANGII-AT1R blocker that reduced edema and degree of injury [87]. These first preclinical data on SARS infection indicated lower ACE2 activity and elevated ANGII locally. In a large animal model of pig, more direct testing of the notion by infusion of ACE2 inhibitor MLN-4760 and low-dose infusion of ANGII that would not be possible in humans yielded higher systolic pulmonary pressure and lower oxygen saturation of blood, and lung perfusion pattern was disturbed, essentially confirming mouse studies [88]. Do observations from human studies confirm these preclinical animal studies? The prediction would be that in patients infected with SARS-CoV-2, depending on load and stage, the virus would engage and decrease ACE2 abundance and/or activity and thus lead to less degradation of ANGII, hence higher concentration of ANGII and lower concentration of Ang1–7.

Several observational studies in humans infected with COVID-19 are published. In this section, we discuss selectively findings from human patient studies that presented data on ACE2 and downstream angiotensin peptides systemically and included a control group, which used protease inhibitors during blood sampling and studies that repeated and confirmed viral detection. Measuring angiotensin peptides is not a trivial task for several reasons. Which is the most appropriate control group? Patients admitted to hospital with other, non-COVID-19, lung infection have been used. Age-matched intensive care patients? Healthy controls not matched for age or sex? All variants have been published. Assay sensitivity and specificity are key factors given low endogenous concentrations (5–50 picomoles/L) of angiotensins and the rapid metabolism *in vivo* and *ex vivo* [89]. Kutz et al. used a validated peptide platform for plasma determinations and observed in patients with COVID-19 lower levels of ANGI, ANGII, Ang1–5, and Ang1–7, and no changes in plasma ACE and ACE2 activities compared with a hospitalized patient group without COVID-19. A strength of this study was that levels of ANGI increased in patients administered ACE inhibitors (ACEi), and levels of ANGII were lower as compared with patients without ACEi. Thus, there was no evidence of increased ANGI and II levels in patients with proven COVID-19 [90]. A retrospective study found that use of ACEi/Angiotensin Receptor Blockers (ARBs) was associated with lower all-cause mortality in patients with COVID-19 with hypertension [91]. In general, use of ACEi/ARBs is no longer as of 2025 considered a risk in patients infected with COVID-19, a topic covered extensively in other review articles which we will not review further here [92–94].

In a prospective observational pilot study of patients at two academic medical centers, angiotensins were determined in patients hospitalized for COVID-19 by validated, high-sensitivity radio-immunoassays after plasma purification and compared with patients hospitalized with acute respiratory illness without COVID-19 [75]. ANGII and Ang1–7 concentrations were measured in the low picomolar range, and there were no differences between patients with COVID-19 and patients with decreased lung function for other reasons. Authors thus did not observe significant differences in RAAS components, and three patients with COVID-19 had significant elevation of Ang1–7, somewhat surprising if ACE2 activity is compromised. Two studies employing commercial ELISAs to detect ANGII in patients with COVID-19 and matched to patients presenting with similar symptoms in the emergency unit [95] and to healthy controls [96] found no differences in ANGII and aldosterone concentrations. A one-center study from Canada used consecutive blood sampling from admitted patients (*n* = 240) of whom 106 required mechanical ventilation or died, and comparisons were made with previously obtained age- and sex-matched healthy controls (*n* = 38). Mass spectrometry was used to quantify angiotensin peptides. At the time of admission (baseline), the levels of ANGII were lower while Ang1–7 was elevated. These changes were associated with lower ACE and no change in ACE2. Authors concluded a lack of overt RAAS activation in COVID-19 regardless of disease severity [76]. A single-center study determined plasma concentrations of sACE2, ANGI, ANGII, and Ang1–7 in 44 patients (30 prolonged-time viral shedders and 14 short-time viral shedders) compared with 15 healthy volunteers. Here, commercial ELISAs were employed and found elevated levels of ANGI and ANGII with no consistent change in Ang1–7 in patients with COVID-19 compared with healthy controls [97]. One study found ANGII to be elevated only in critically ill patients in intensive care and reported no change in mild or even in severe COVID-19 infection [98]. In summary, a consistent picture emerges across countries, different assay types, and age of individuals where circulating levels of ANGI and II are not increased, rather in some reports decreased, in patients with mild and severe COVID-19, while critically ill intensive care patients have elevated levels. No consistent change in Ang1–7 in patients with COVID-19 can be extracted at present. Therefore, differences in ANGII and Ang1–7 levels are less likely to explain more negative outcomes in elderly males vs. females and younger adults.

### Summary and conclusion

ACE2 and TMPRSS2 mRNA expression and protein levels are highest in the GI tract, kidney, and reproductive organs, while there are comparatively much lower levels in the lungs. Although selected organs co-express the proteins, the co-localization of the two proteins in humans is limited, and most data are from *in vitro* studies. While ACE2 is more abundant in female than male kidneys from persons under 50 years and is sensitive to estrogen, TMPRSS2 responds to androgens and is more abundant in the male reproductive organs. However, there are no documented sex-specific changes in organ distribution or abundance of ACE2 and TMPRSS2 that occur with age in adults that could increase susceptibility to SARS-CoV-2 infection. Although SARS-CoV-2 hijacks ACE2 to invade cells, this is not reflected in plasma, while urine ACE2 is increased severalfold (more than 100 times). In patients with COVID-19, there is no consistent change in circulating ACE and ACE2 activity and in plasma concentrations of ANGII and Ang1-7 in mild-to-serious infection. Increased levels of ANGII are reported in the most critically ill patients but may not be COVID-19 disease-specific. In conclusion, the quantitative organ distribution and sex dependence of ACE2 and TMPRSS2 levels may explain the primary airborne route for SARS-CoV-2 infection and lower infection rate in children but do not explain the more serious disease in elderly males.

**Perspectives**

- COVID-19 engages with angiotensin-converting enzyme 2 (ACE2), which is part of RAAS and is associated with significantly higher morbidity and mortality in the elderly and in male sex. It is clinically relevant to consider the organ distribution and abundance of ACE2 and co-factor transmembrane serine protease 2 (TMPRSS2) with age and sex and how COVID-19 affects enzyme activity and subsequently peptide products ANGII and Ang1–7.
- ACE2 and TMPRSS2 co-localization in oral and airway mucosa accounts for susceptibility via the airborne route of infection, while kidneys, GI tract, and reproductive epithelia show much higher ACE2 levels but with lower susceptibility. There are no major changes in circulating angiotensins with mild-to-serious COVID-19, and common antihypertensive drugs such as ACEi/ARBs are not associated with negative outcomes. In the elderly, ACE2 shows no difference in abundance between sexes that can account for higher morbidity and mortality in males.
- Key unanswered questions for future directions
  - Does plasma angiotensin-converting enzyme 2 (ACE2) changes have a significant role (positive/negative) in the pathophysiology and susceptibility to COVID-19 disease?
  - Which enzymes beyond transmembrane serine protease 2 are important in humans *in vivo* for susceptibility to COVID-19?
  - Is a high degree of loss of ACE2 from kidney epithelia into urine in severe COVID-19 predicting negative kidney outcome?
  - Is the loss of the ACE2/Ang1–7 axis significant for the progression of severe COVID-19 disease systemically?
  - Can insight into the disease mechanisms for SARS-CoV-2 obtained in cells and preclinical studies be translated into the critically ill COVID-19 patients employing e.g. infusion of soluble recombinant ACE2, Ang1–7, AT2 receptor and/or Mas receptor agonists and nitric oxide?

## Abbreviations

ACE2: Angiotensin-Converting-Enzyme 2
ACE: Angiotensin-Converting-Enzyme
ACEi: Angiotensin- 19 Converting-Enzyme inhibitors
ANGI: Angiotensin I
ANGII: Angiotensin II
ARBs: Angiotensin Receptor Blockers
AT2: alveolar type 2
AT1R: angiotensin type 1 receptor
AT2R: angiotensin type 2 receptor
COVID- 19: coronavirus-disease – 19
Cat B/L: Cathepsin B/L
ECM: extracellular matrix
GI: Gastrointestinal
RAAS: Renin-Angiotensin- Aldosterone-System
TMPRSS2: transmembrane protease serine 2
sACE2: soluble ACE2
